# Weighted Brain Network Metrics for Decoding Action Intention Understanding Based on EEG

**DOI:** 10.3389/fnhum.2020.00232

**Published:** 2020-07-02

**Authors:** Xingliang Xiong, Zhenhua Yu, Tian Ma, Ning Luo, Haixian Wang, Xuesong Lu, Hui Fan

**Affiliations:** ^1^Key Laboratory of Child Development and Learning Science of Ministry of Education, School of Biological Science & Medical Engineering, Southeast University, Nanjing, China; ^2^College of Computer Science and Technology, Xi'an University of Science and Technology, Xi'an, China; ^3^Institute of Software, Chinese Academy of Sciences, Beijing, China; ^4^Department of Rehabilitation, Zhongda Hospital, Southeast University, Nanjing, China; ^5^Co-innovation Center of Shandong Colleges and Universities: Future Intelligent Computing, Shandong Technology and Business University, Yantai, China

**Keywords:** action intention understanding, phase lag index, weighted network metric, classification accuracy, mirror neuron system, mentalizing system

## Abstract

**Background:** Understanding the action intentions of others is important for social and human-robot interactions. Recently, many state-of-the-art approaches have been proposed for decoding action intention understanding. Although these methods have some advantages, it is still necessary to design other tools that can more efficiently classify the action intention understanding signals.

**New Method:** Based on EEG, we first applied phase lag index (PLI) and weighted phase lag index (WPLI) to construct functional connectivity matrices in five frequency bands and 63 micro-time windows, then calculated nine graph metrics from these matrices and subsequently used the network metrics as features to classify different brain signals related to action intention understanding.

**Results:** Compared with the single methods (PLI or WPLI), the combination method (PLI+WPLI) demonstrates some overwhelming victories. Most of the average classification accuracies exceed 70%, and some of them approach 80%. In statistical tests of brain network, many significantly different edges appear in the frontal, occipital, parietal, and temporal regions.

**Conclusions:** Weighted brain networks can effectively retain data information. The integrated method proposed in this study is extremely effective for investigating action intention understanding. Both the mirror neuron and mentalizing systems participate as collaborators in the process of action intention understanding.

## Introduction

Understanding others' intentions from their actions is thought to be an essential part of social interaction (Satpute et al., 2005; Cacippo et al., 2010; Ortigue et al., 2010; Catmur, 2015; Pereira et al., 2017; Pomiechowska and Csibra, 2017) and to play an important role in children's physiological growth and language learning (Fogassi et al., 2005; Ouden-Den et al., 2005; Brune and Woodward, 2007; Kaschak et al., 2010; Casteel, 2011; Isoda, 2016). However, the complex mechanism underlying action intention understanding has not been thoroughly decoded (Virji-Babul et al., 2010; Bonini et al., 2013; Catmur, 2015; Tidoni and Candidi, 2016; Cacioppo et al., 2017; Pomiechowska and Csibra, 2017; Cole and Barraclough, 2018; Cole et al., 2018). Exploring these neuronal mechanisms would be very useful for application into research on intelligent human-robot interaction (HRI) and brain-computer interface (BCI) (Zhang et al., 2015; Liu et al., 2017; Ofner et al., 2017).

Machine learning is an extremely important tool that is widely applied in biomedical engineering (Dindo et al., 2017; Mcfarland and Wolpaw, 2017; Ofner et al., 2017; Pereira et al., 2017; Bockbrader et al., 2018). For studying action intention understanding, good classification accuracy is one of the most critical factors (Dindo et al., 2017; Ofner et al., 2017; Bockbrader et al., 2018). In recent years, many researchers have performed numerous experiments with machine learning, but most of the classification results are unsatisfactory (Zhang et al., 2015, 2017; Liu et al., 2017; Pereira et al., 2017). After investigating these previous studies, we determined two important reasons for these poor classification results. One is the extraction of useless features, and the other is the selection of a small number of samples for training classifier model. For feature extraction, many methods (e.g., time domain and frequency domain analyses) have been introduced to neuroscience (Ortigue et al., 2010; Carter et al., 2011; Ge et al., 2017; Liu et al., 2017; Pereira et al., 2017; Pomiechowska and Csibra, 2017; Zhang et al., 2017), but these methods do not perform sufficiently well in terms of the actual results. For sample collection, due to limitations in recruiting participants, it is very difficult to obtain a large number of samples (Zhang et al., 2015, 2017; Liu et al., 2017; Pereira et al., 2017).

In view of the above introduction, we implement classification tasks for EEG signals related to action intention understanding from the perspectives of both feature extraction and sample collection in this study. To extract useful features, we first aim to obtain reliable time series in the source space by sLORETA. And then, we use the phase lag index (PLI) (Stam et al., 2007) and weighted phase lag index (WPLI) (Vinck et al., 2011) to construct dynamic brain networks in multiple micro-time windows and specific frequency bands. It is worth mentioning that many other methods (synchronization likelihood Stam and Dijk, 2002, phase lock value Lachaux et al., 1999, Pearson correlation, etc.) have the weakness of volume conduction effect when computing the brain network based on EEG signals (Stam et al., 2007; Niso et al., 2013), whereas both the PLI and WPLI methods can solve this problem well (Stam et al., 2007; Vinck et al., 2011). In recent studies about action intention understanding, Zhang et al. (2017) obtain better experimental results with the WPLI than the results of Zhang et al. (2015) that based on phase synchronization and Pearson correlation. Hence, we naturally think that select the PLI and WPLI to construct brain network. Hard back to the subject, we finally calculate a number of graph complexity measures as the classification features. Notably, many studies attach great importance to using a binary network to decode brain signals related to action intention understanding (Zhang et al., 2015, 2017). However, others argue that network thresholding easily results in the loss of some useful information (Phillips et al., 2015; Ahmadlou and Adeli, 2017). This is mainly because weighted brain networks are very sensitive to the threshold. Recently, Ahmadlou and Adeli (2017) proposed a new approach that adopted two weighted undirected graph complexity measures to study autism and aging issues and achieved satisfactory statistical results. Considering these facts, it is naturally thought that a weighted brain network can be used to decode action intention understanding. To collect more a larger number of samples, we converted each subject in a certain class of brain signals into two subjects by constructing two brain networks, one from the PLI and the other from the WPLI. Because our final goal is to classify the different kinds of brain signals related to action intention understanding, the transformation is feasible.

Our method is mainly based on the state-of-the-art dynamic time-frequency brain network technique, which has numerous advantages. For instance, it can consider both time and frequency feature information, locate activated brain areas, and discover potential topological relationships among regions of interest (ROIs). These merits can help us decode action intention understanding more comprehensively than single time or frequency analyses (Rubinov and Sporns, 2010; Zhang et al., 2015, 2017; Vecchio et al., 2017; Cignetti et al., 2018). The scheme of sample reconstruction is very important for this study, as it improves the classification accuracy efficiently, especially for the classification of similar action intention stimuli.

## Materials and Methods

### Subjects

A total of 30 healthy subjects were recruited for EEG data acquisition. All participants did not use any prescribed medication, and they also did not have any neurological disease. Before the start of the experiments, they were asked to read and sign an informed consent form. When finished with the tasks, all participants received monetary compensation. After deleting the data from 5 subjects that had been heavily degraded by bad channels, we finally collected EEG data from 25 subjects (17 males, 8 females; age 19–25 years, mean ± SD: 22.96 ± 1.54; all right-handed). This study was supported by the Academic Committee of the School of Biological Sciences and Medical Engineering, Southeast University, China.

### Experimental Paradigm

For data acquisition, all participants were told to view three hand-cup interaction pictures that were shown on a computer monitor with E-prime 2.0. The subjects were asked to only silently judge the intention of the hand-cup interaction. The three action intentions were drinking water, moving the cup and simply touching the cup. This design comes from the research of Ortigue et al. (2010).

**Figure 1:**
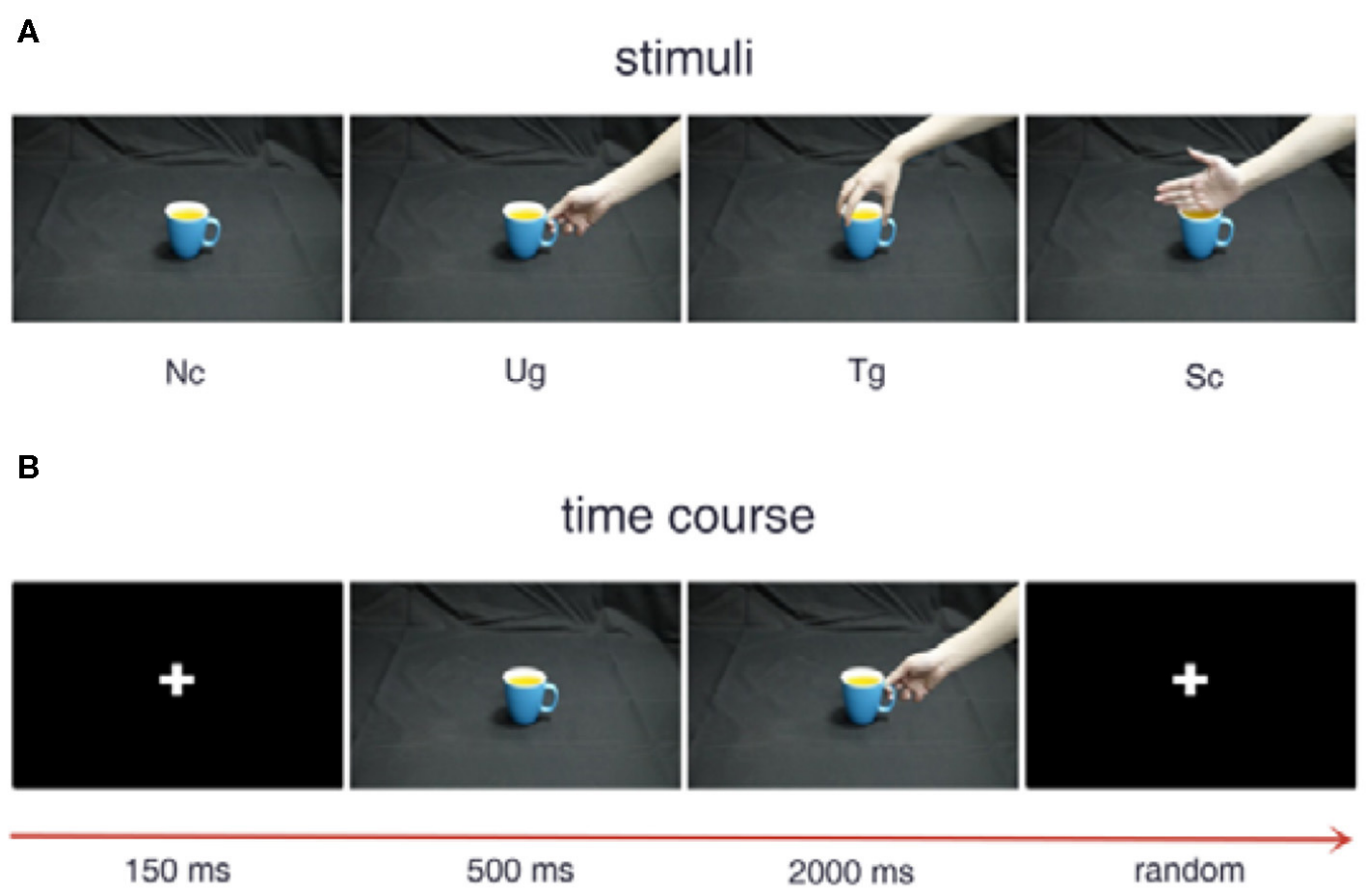
Experimental stimuli and procedure. **(A)** Examples of the different stimuli. **(B)** Stimulus presentation in a trial.

Figure 1 shows the experimental stimuli and procedure. The three kinds of hand-cup interaction stimuli are presented in Figure 1A, where Ug (use grip) denotes a hand that is grasping a cup with the intention of drinking water, Tg (transport grip) represents a hand that is grasping a cup with the intention of moving it, and Sc (simple contact) denotes a hand that is touching a cup without any clear intention. Figure 1B illustrates the experimental stimuli presented in a trial, which are shown sequentially over the indicated time course. During the experiment, a white cross first appeared in the center of the screen for 150 ms. Then, a cup was shown on the screen for 500 ms. After the cup disappeared, a hand-cup interaction stimulus was displayed on the screen for 2,000 ms. Once the hand-cup interaction appeared on the screen, the subjects were to immediately and silently judge the actor's intention. Before the next trial, the cross was shown again for a random time that varies from 1,000 to 2,000 ms.

All participants underwent a 12-trial practice session before the formal experiment. Across all participants, the practice session lasted for an average of approximately 24 min. To alleviate visual fatigue caused by repeated experimentation, we presented the cups with a color chosen randomly among seven different colors for each trial. Because before formal EEG signal acquisition experiment, all participants are informed that they only need to judge what is the intention of the actor's gesture, and the color of the cup has nothing to do with the performer's intention. Thereupon, the actor's gesture is more important in the stimulus procedure when comparing with the factor of cup color. The mixture of color variables does not affect the classification effect. In this research, each action intention condition was shown in 98 trials.

### Data Collection

The signals were obtained by using 64 AgCl electrodes positioned with the international 10–20 system. We set the sampling rate to 500 Hz. The M1 electrode served as a reference electrode and was placed on the left mastoid, and the GND electrode served as a ground electrode and was placed at the center of the frontal scalp. Additionally, four other channels (HEOR, VEOU, HEOL, and VEOL) were placed around the eyes of the participants to record electrooculographic (EOG) signals. All the data collection tasks were carried out in Neuroscan 4.3.

### Preprocessing the Raw Data

To obtain clean data, we applied two popular neuroscience computer programs, Neuroscan 4.3 and EEGLAB 14.0 (Arnaud and Scott, 2004), to implement several preprocessing steps for the raw EEG signals.

Based previous experimental experience, it remains difficult to clean EEG data with ICA in EEGLAB. Hence, we applied ocular processing to replace ICA in Neuroscan. Given that the mastoid reference is active and effective in detecting somatosensory evoked potentials, we re-referenced data from the unilateral mastoid electrode (M1) to the average of the bilateral mastoid electrodes (M1, M2). As with the ocular processing, the re-referencing was also conducted in Neuroscan.

**Figure 2:**
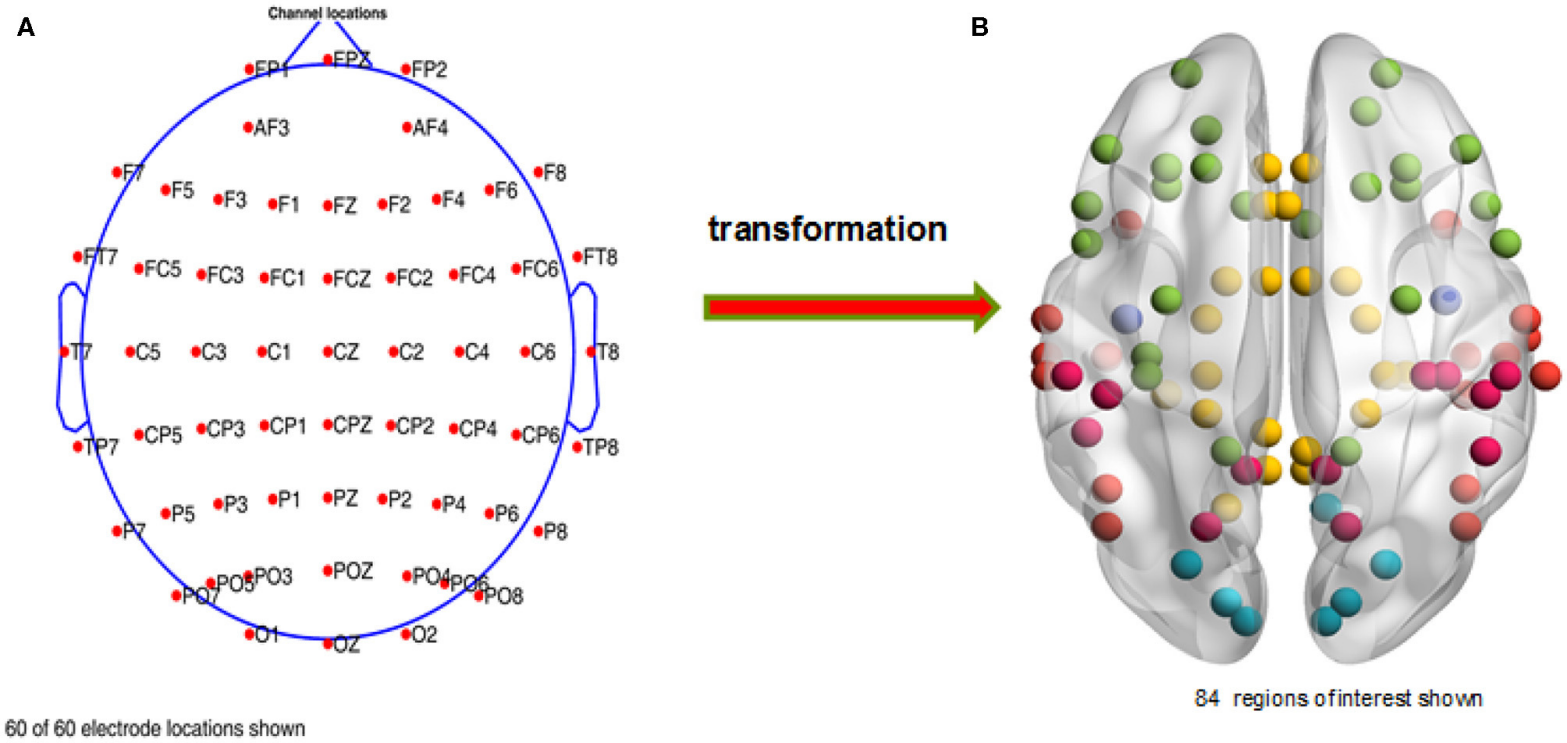
Distribution of the chosen channels and regions of interest. **(A)** The retained electrodes cover most of the domains on the participant's scalp, i.e., frontal, occipital, left, right, and central. **(B)** A total of 84 regions of interest are selected, which are mainly distributed in the temporal, limbic, frontal, occipital, and parietal lobes and the sub-lobar regions.

After finishing the ocular processing and re-referencing, we chose the required electrodes (see Figure 2A, a total of 60 channels were preserved for each subject) with EEGLAB. Then, we applied the Basic FIR filter in EEGLAB to extract the full frequency band (1–30 Hz) data. And then, we segmented the EEG data with event types in discrete time windows (−0.65 s to 2.5 s) and subtracted the baseline signal, obtained from −0.65 to 0 s. In the end, we deleted artifacts with a threshold range that varied from −75 to 75 μ*v*; i.e., voltage signals between −75 and 75 μ*v* were retained and otherwise removed as artifacts. A total of 679 trials were deleted, and an average of 267 trials were retained for each subject in this study.

### Construction of a Functional Connectivity Matrix

For constructing a functional connectivity matrix, many algorithms have been proposed by researchers in recent years (Schreiber, 2000; Baccalá and Sameshima, 2001; Nolte et al., 2004; Niso et al., 2013). However, most of these methods are unable to contend with volume conduction. To effectively solve this problem, Stam et al. (2007) proposed the phase lag index (PLI).

(1) $$\begin{array}{l} PLI_{AB}=\left|\frac{1}{N}\overset{N}{\underset{n=1}{\sum }}sign\left(\Delta \theta \left(t_{n}\right)\right)\right| \end{array}$$

where Δθ denotes the instantaneous phase difference between time series *A*(*t*) and *B*(*t*) at the *nth* sample time point. We obtained the instantaneous phase by adopting the Hilbert transform.

However, across many experiments, people find that the PLI algorithm still has some shortcomings. A significant weakness of the PLI is that it is easily affected by noise. Based on the PLI, researchers designed a reinforcement method named the weighted phase lag index (WPLI) (Vinck et al., 2011). Let $\tilde{S}\left(X\right)$ be the imaginary component of the cross-spectrum between time series *A*(*t*) and *B*(*t*). Then, the WPLI is defined by:

(2) $$\begin{array}{l} WPLI_{AB}=\frac{\left|\langle \tilde{S}\left(X\right)\rangle \right|}{\langle \left|\tilde{S}\left(X\right)\right|\rangle }=\frac{\left|\langle |\tilde{S}\left(X\right)|sign\left(\tilde{S}\left(X\right)\right)\rangle \right|}{\langle \left|\tilde{S}\left(X\right)\right|\rangle } \end{array}$$

where 〈▪〉 and |▪| denote the mean and absolute value operations, respectively, and *sign* is the signum function.

**Figure 3:**
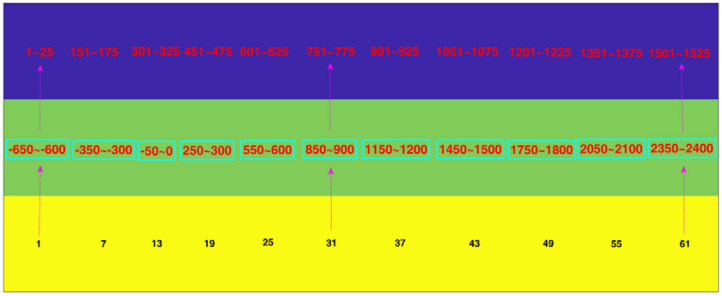
The corresponding relation among sample points, time ranges, and time window numbers. The blue, green, and yellow rows denote the sample points, time ranges and time window numbers, respectively. For example, the digit “1” in the yellow row corresponds to “−650 to −600” in the green row and “1 to 25” in the blue row. That is, the first time window is from −650 ms to −600 ms, which contains the 1st to the 25th time points.

In order to carry out whole brain research, we first transformed the time series of the 60 scalp electrodes into 84 brain regions of interest in the source space (see Figure 2B) with sLORETA, and then used the PLI and WPLI to construct functional connectivity matrices in 5 frequency bands and 63 time windows. Notably, we applied the filter of sLORETA to extract the specific frequency band (1–4, 4–8, 8–13, and 13–30 Hz; i.e., delta, theta, alpha, and beta sub-bands, respectively) data. Because many experiments in the following sections involve time windows, we will illustrate the corresponding relations among the sample points, time ranges and time window numbers (see Figure 3).

As for the brain network, each brain region is defined as a network node, and the functional connection between any two regions denotes an edge of the network in this study.

### Network Metrics

After obtaining the functional connectivity matrices that were computed from the PLI and WPLI, we directly calculated the network metrics for these matrices. In this study, we applied nine graph measures-graph index complexity ***Cr*** (Kim and Wilhelm, 2008), graph density ***GD*** (Gomezpilar et al., 2017), Shannon graph complexity ***SGC*** (Gomezpilar et al., 2017), average neighbor degree ***K*** (Barrat et al., 2004; Rubinov and Sporns, 2010), efficiency complexity ***Ce*** (Kim and Wilhelm, 2008), global efficiency ***Ge*** (Latora and Marchiori, 2001; Rubinov and Sporns, 2010), clustering coefficient ***C*** (Onnela et al., 2005), characteristic path length ***L*** (Watts and Strogatz, 1998), and small-world ***SW*** (Humphries and Gurney, 2008; Rubinov and Sporns, 2010)-to decode action intention understanding.

**Figure 4:**
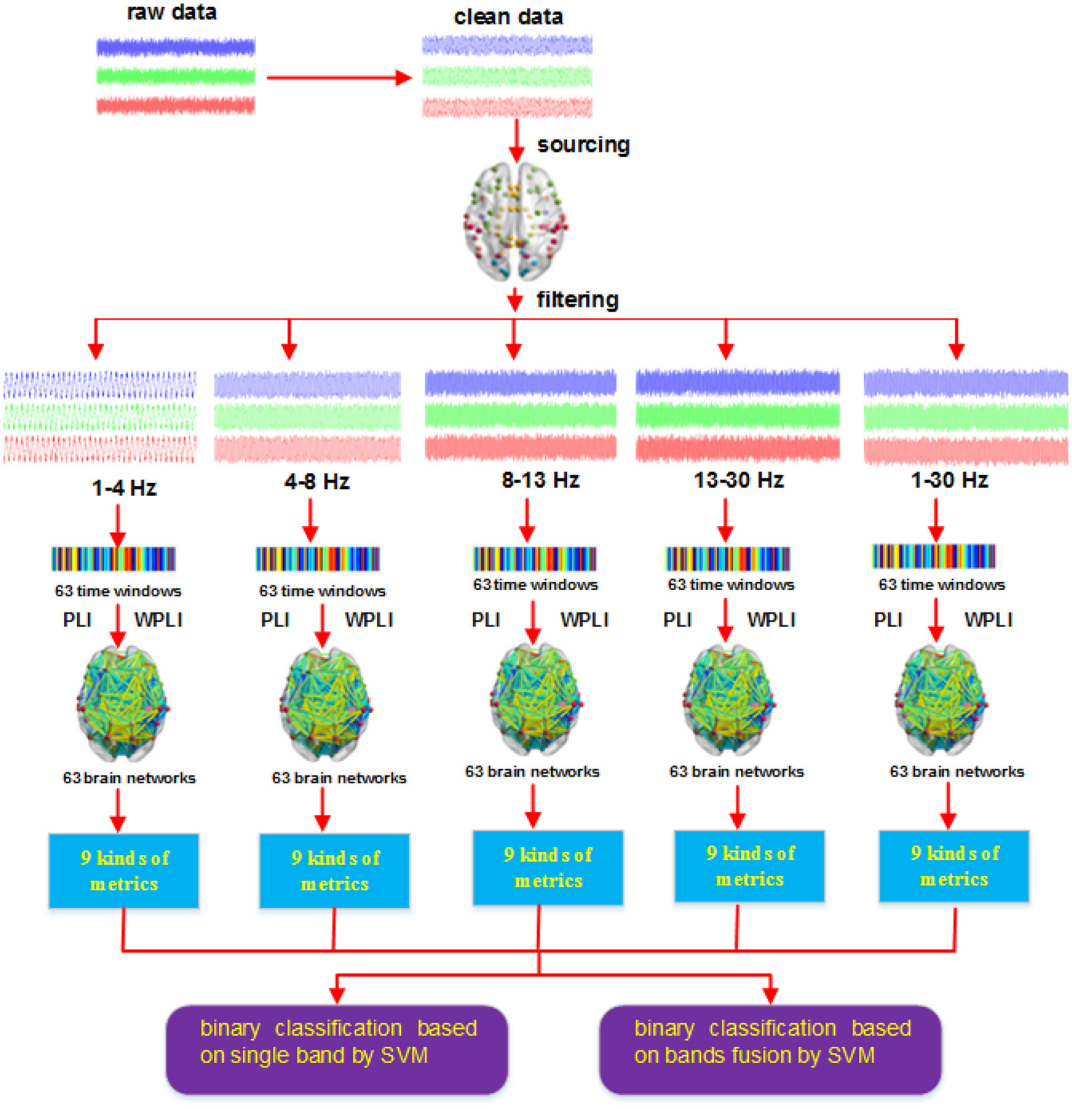
Procedures of the new method.

After we obtained the network metrics, we then applied them to decode action intention understanding, include classifying different intentions and exploring how brain activity changed with time. Figure 4 shows the steps of our method. The key point of our new method is that combining the two methods (PLI and WPLI) for constructing the brain network increases the number of training samples and features, which is different from the single methods that only use PLI or WPLI to extract features. Our binary classification task is performed at the group level. Each stimulus has 25 samples (the number of participants), 9 graph metrics and 63 time windows. Hence, for a single method (PLI or WPLI) using the fusion time windows, the dimensions of the dataset are 50 × 567 (50 samples, 567 features) for each frequency band and 50 × 2,835 (50 samples, 2,835 features) for the fusion bands. For the new method (PLI+WPLI) using the fusion time windows, the dimensions of the dataset are 100 × 567 (100 samples, 567 features) for each frequency band and 100 × 2,835 (100 samples, 2,835 features) for the fusion bands. Similarly, for the dynamic time windows, we obtain the dataset dimensions: 50 × 9 for PLI or WPLI for single bands, 50 × 45 for PLI or WPLI for the fusion bands, 100 × 9 for the new method for single bands and 100 × 45 for the new method for the fusion bands.

## Results

In this section, we describe the experimental results obtained mainly by using the weighted brain network metrics. Our experimental results consist of four parts: time series analysis, feature selection, binary classification and brain network analysis. Details of the four parts are given in the following subsections.

### Time Series Analysis

**Figure 5:**
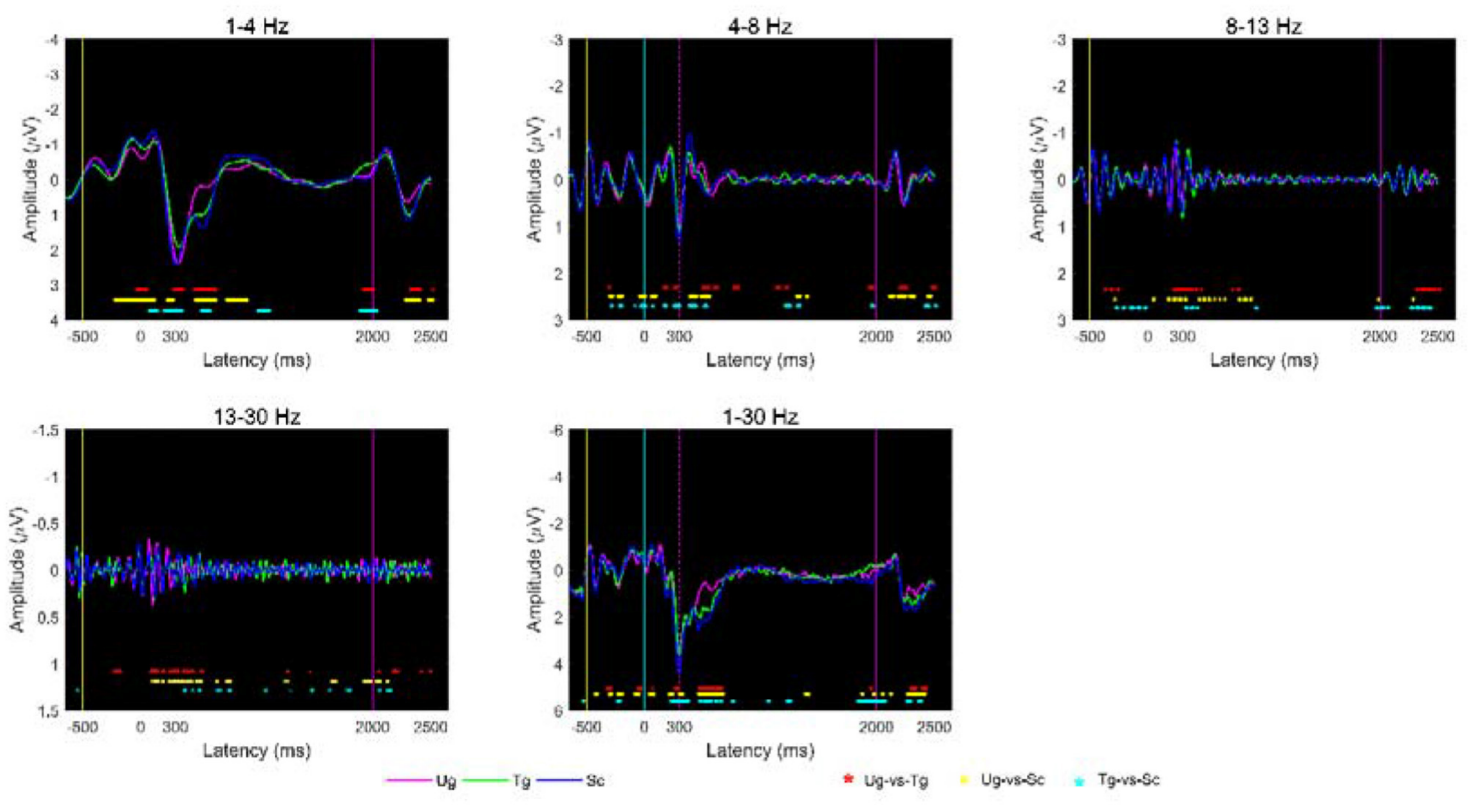
Average ERP at the group-level under different stimulus conditions. The yellow, cyan, and magenta vertical lines represent the end time of the symbol “+,” cup, and hand-cup interaction presentations, respectively. The magenta, blue, and green curves denote the average amplitudes across all subjects for the three stimulus conditions Ug, Tg, and Sc, respectively. The magenta dotted line denotes the start of the P300 component. The red, yellow and cyan “^*^” symbols at the bottom of the plots denote *p* < 0.05 according to *t*-tests, where the colors, respectively, correspond to Ug-vs-Tg, Ug-vs-Sc, and Tg-vs-Sc.

To determine whether differences exist under different stimuli, we analyzed the voltage signals from 650 milliseconds before to 2,500 milliseconds after formal stimulation. Figure 5 shows the average ERPs of the three hand-cup interactions across all subjects and all trials. As indicated by *t*-tests (*p* < 0.05), we can see that the three ERPs are often significantly different between −650 and 2,500 ms; in particular, the amplitudes around P300 (~0–600 ms) of the Ug, Tg, and Sc ERPs are extremely different. Additionally, we can also see that each kind of ERP has an exact and significant P300 component (see the magenta dotted line).

### Feature Selection

**Figure 6:**
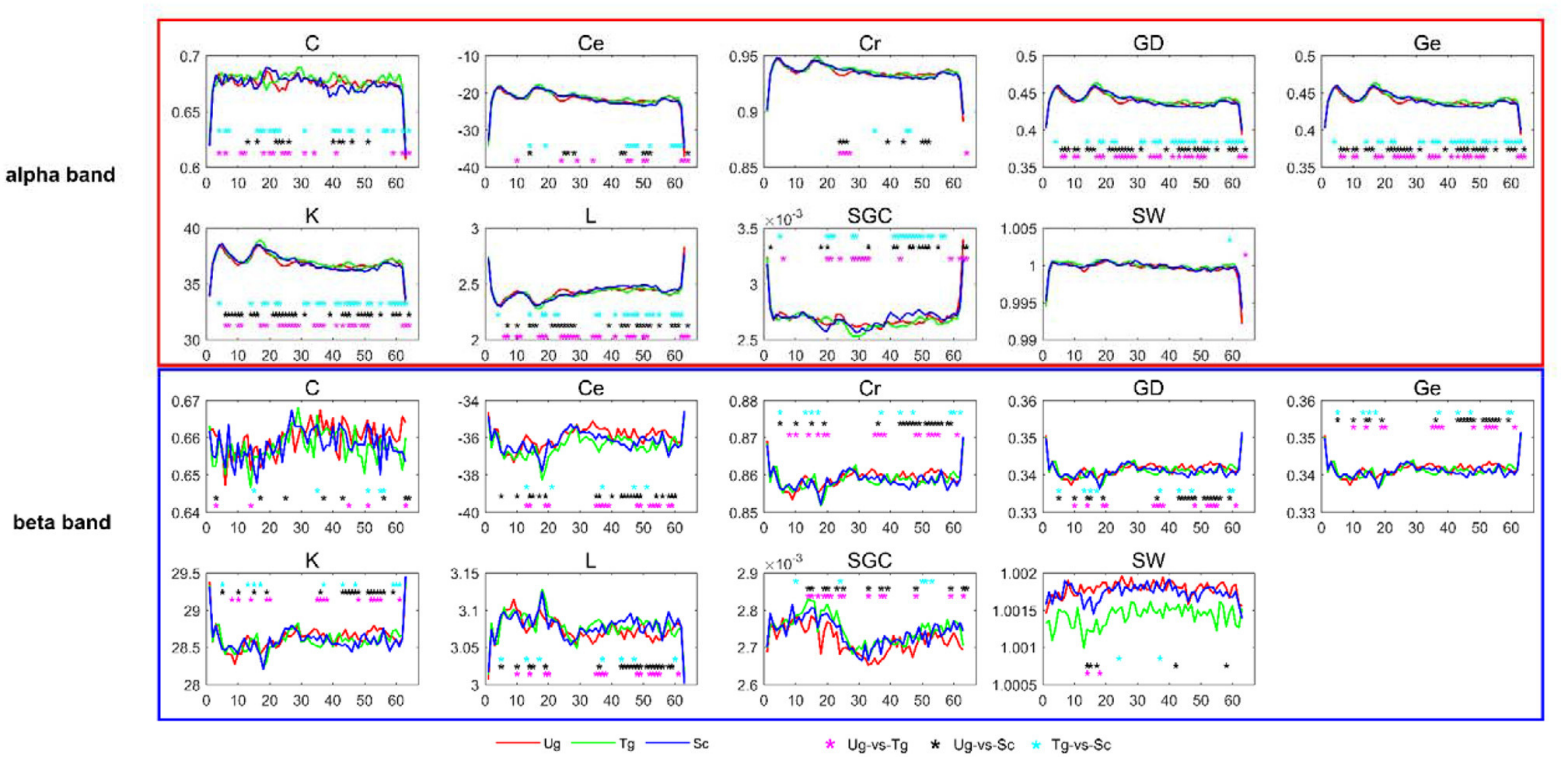
*T*-tests of brain network metrics. The red, green, and blue curves denote the values of the metrics under the Ug, Tg, and Sc conditions, respectively. The cyan, black and magenta “^*^” symbols denote significant differences determined by *t*-tests (*p* < 0.05) for the Tg-vs-Sc, Ug-vs-Sc, and Ug-vs-Tg paired intentions, respectively. The horizontal and vertical axes represent the time window number and graph metric value, respectively.

In this study, we selected the weighted brain network metrics to use as classification features. As introduced previously, all the functional connectivity matrices were constructed with the PLI and WPLI, and the nine metrics-***Cr***, ***GD***, ***SGC***, ***K***, ***Ce***, ***Ge***, ***C***, ***L***, and ***SW***-were computed in the delta, theta, alpha, beta and full frequency bands. Figure 6 shows the dynamic changes in the nine metrics in both the alpha and beta bands. After applying *t*-tests (*p* < 0.05), we can see that each metric has a different effect, with some reflecting greater differences than others between the two specific frequency bands. For instance, ***GD***, ***Ge***, ***K***, and ***L*** are better than ***Cr***, ***SGC***, ***Ce***, ***C***, and ***SW*** in the alpha band. Additionally, we can also see that most of the metrics can effectively reflect the differences of the paired intentions in many time windows, especially in the time windows around some specific components, such as C100, N170, and P300.

### Classification Accuracy

In this study, we adopted weighted brain network (PLI and WPLI) metrics to carry out action intention understanding classifications. Binary classification, i.e., a one-vs.-one strategy, was implemented. We designed three pairwise action intention understanding tasks, “Ug-vs-Tg,” “Tg-vs-Sc,” and “Ug-vs-Sc.” The classical classifier, SVM, was chosen for data classification. For the parameters of the classifier, we selected a polynomial kernel function with the order set as 1. For each classification task, we used 5-fold cross-validation to avoid overfitting, and each 5-fold cross-validation was implemented 50 times. The classification results are the means of the 50 implementations of the 5-fold cross-validation.

**Figure 7:**
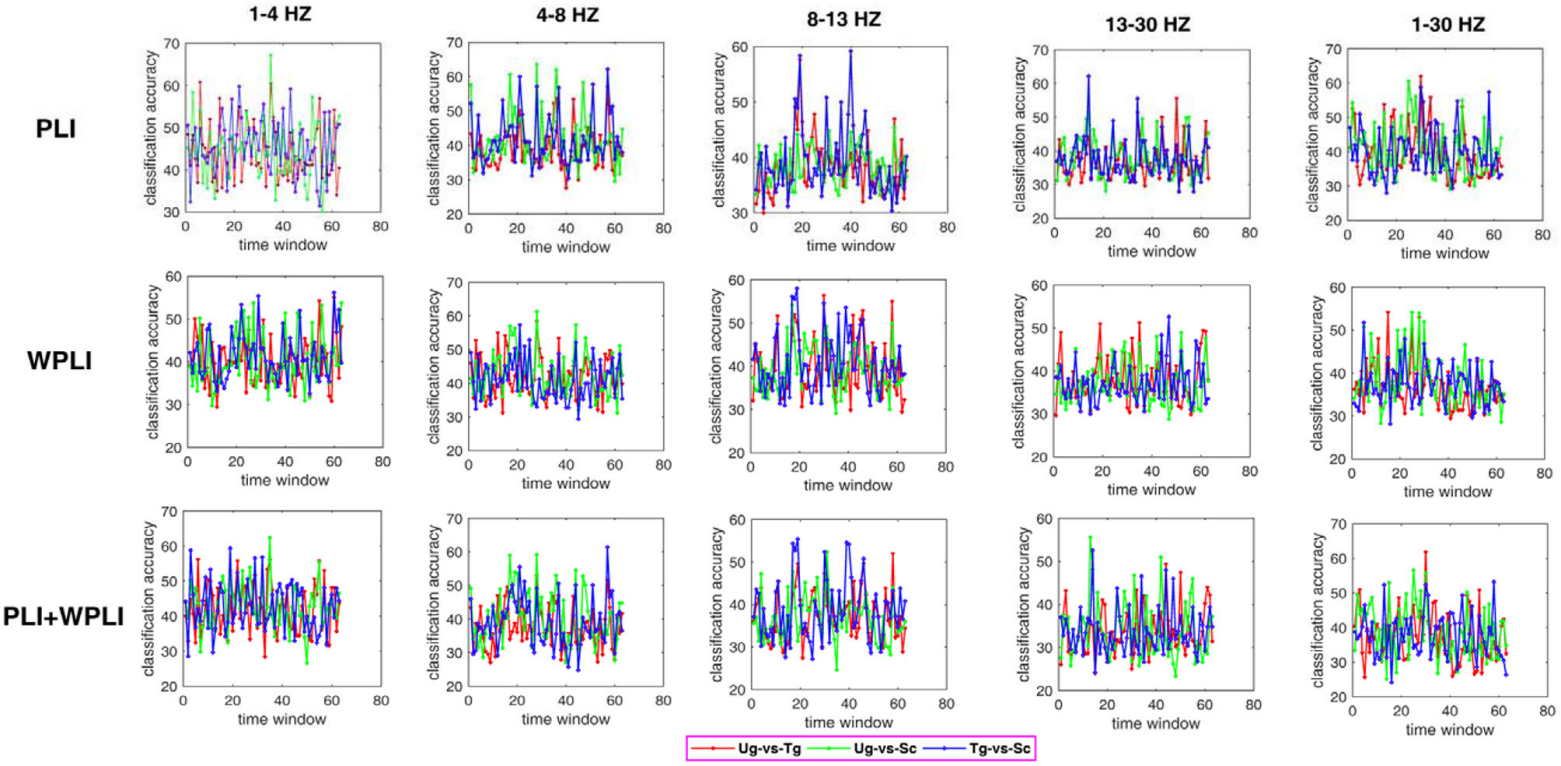
Classification accuracies in different time windows. The subfigure 1–5 are the classification results that applied graph metrics which obtained by using the PLI to construct brain network in five bands, subfigure 6–10 are the results obtained by WPLI on the same conditions as 1–5, and subfigure 11–15 are the results that calculated by combining both PLI and WPLI.

**Figure 8:**
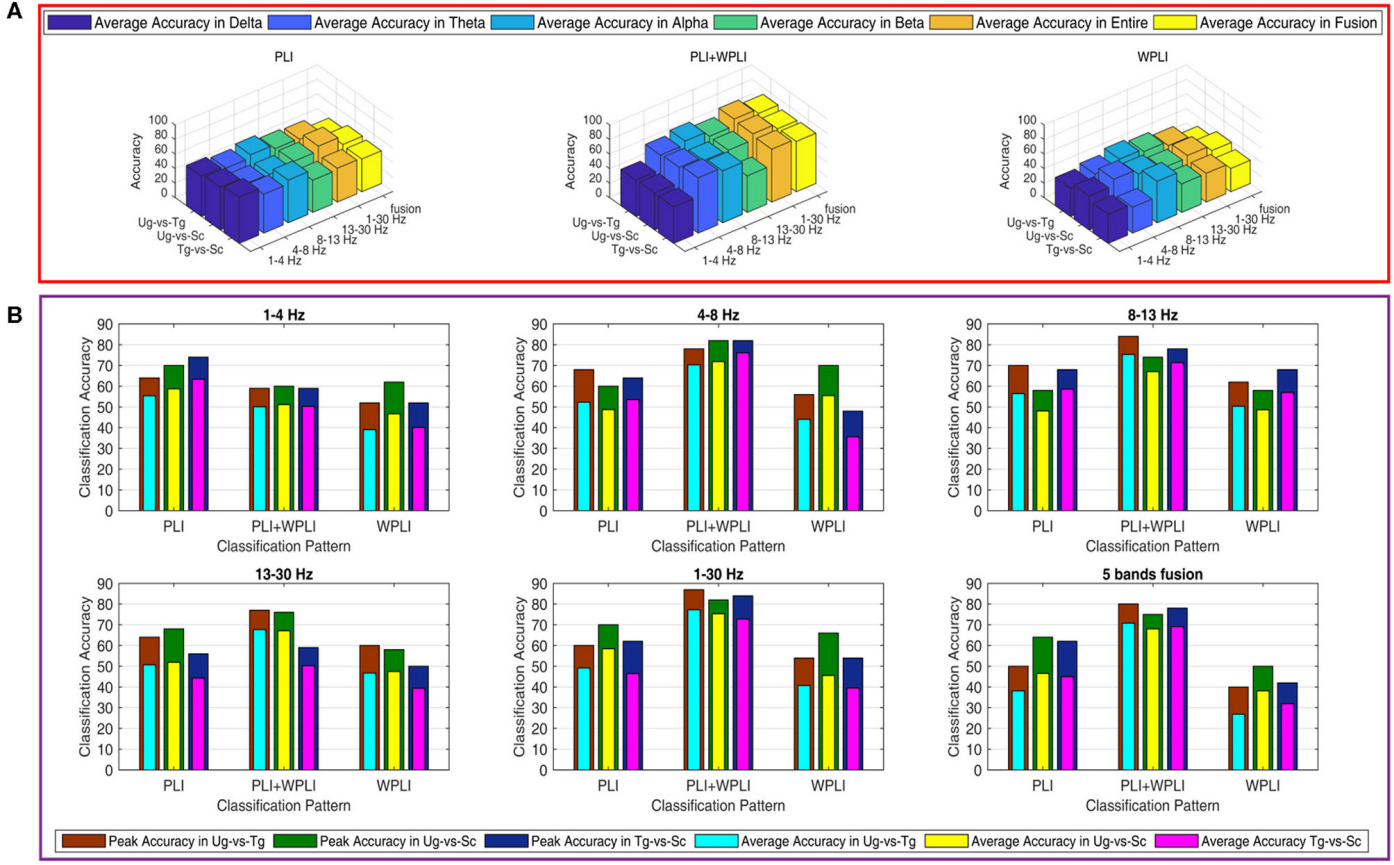
Classification accuracies in fusion time windows. **(A)** Comparison in a special method (PLI, WPLI, or PLI+WPLI). The bar denotes the average accuracy on a special combination condition (frequency band and pair-intentions). **(B)** Comparison among the three methods (PLI, WPLI, and PLI+WPLI). The external layer bar is the maximum classification accuracy and the inner layer bar represents the average classification accuracy.

Figure 7 shows the classification results in different time windows. From subfigures 1–15, we can see that there are some peak accuracies around the specific components, e.g., C100, N170, and P300, especially in the alpha and beta frequency bands. Additionally, we can also see that the classification accuracies for both “Tg-vs-Sc” and “Ug-vs-Sc” are better than for “Ug-vs-Tg” in most cases. However, except for a few time windows, the classification results in all other time windows are unsatisfactory. Most of the classification accuracies are lower than 60%. For the three methods, PLI, WPLI, and PLI+WPLI, there is no obvious advantage to any one of them. Figure 8 demonstrates the classification accuracies in the fusion time windows (i.e., the merging of the brain network metric features from the 63 time windows into a large dataset). As shown in Figure 8A, the PLI, PLI+WPLI, and WPLI methods have different average accuracies for the different frequency bands. The low frequency band performance is the worst for both WPLI and our new method. Notably, all methods perform well on the alpha band. Additionally, the Ug-vs-Tg classification reaches levels that are not worse than those of both Ug-vs-Sc and Tg-vs-Sc. Figure 8B shows the comparisons of the classification accuracies among the three different methods. From the six subpictures in Figure 8, we can see that our new method performs better than the other two methods except for the low frequency band, where the new method performs worse than PLI. In terms of concrete results, most of the average classification accuracies of the novel method-are over 60%, with some approaching 80%, while some of the maximum classification accuracies approach 90%; for example, see the results for 4–8, 8–13, and 1–30 Hz. More details on the classification accuracy are shown in Table 1.

**Table 1:** Five estimation measures under different conditions.

**Frequency band**	**Classification strategy**	**Method**	**Maximum accuracy**	**Average accuracy**	**Standard deviation**	**Sensitivity**	**Specificity**
1–4 Hz	Ug-vs-Tg	PLI	64.00	55.36	14.43	55.66	57.71
		PLI+WPLI	59.00	50.04	10.74	53.54	47.54
		WPLI	52.00	39.00	13.53	42.03	38.79
	Ug-vs-Sc	PLI	70.00	58.72	12.60	61.69	58.43
		PLI+WPLI	60.00	51.12	9.74	55.56	47.40
		WPLI	62.00	46.72	15.05	50.07	47.10
	Tg-vs-Sc	PLI	74.00	63.32	12.52	63.70	65.87
		PLI+WPLI	59.00	50.26	10.41	47.89	53.67
		WPLI	52.00	40.16	13.58	42.06	40.96
4–8 Hz	Ug-vs-Tg	PLI	68.00	52.28	13.70	58.73	48.37
		PLI+WPLI	78.00	70.24	8.86	71.74	70.04
		WPLI	56.00	44.04	12.95	47.47	43.50
	Ug-vs-Sc	PLI	60.00	48.68	14.07	49.30	51.75
		PLI+WPLI	82.00	71.86	8.72	72.09	72.73
		WPLI	70.00	55.40	14.15	61.85	52.50
	Tg-vs-Sc	PLI	64.00	53.48	14.33	53.40	55.73
		PLI+WPLI	82.00	76.06	8.85	76.50	76.29
		WPLI	48.00	35.56	13.24	37.52	35.59
8–13 Hz	Ug-vs-Tg	PLI	70.00	56.44	14.81	57.78	56.53
		PLI+WPLI	84.00	75.24	8.82	77.65	73.90
		WPLI	62.00	50.36	14.66	46.87	55.17
	Ug-vs-Sc	PLI	58.00	48.04	13.93	47.54	50.74
		PLI+WPLI	74.00	66.96	9.47	69.46	65.83
		WPLI	58.00	48.56	13.44	48.03	51.94
	Tg-vs-Sc	PLI	68.00	58.64	14.03	59.33	60.03
		PLI+WPLI	78.00	71.32	7.97	71.78	72.75
		WPLI	68.00	56.96	13.14	55.47	60.24
13–30 Hz	Ug-vs-Tg	PLI	64.00	50.68	13.44	52.42	51.50
		PLI+WPLI	77.00	67.68	10.84	67.67	68.50
		WPLI	60.00	46.72	13.87	46.91	48.88
	Ug-vs-Sc	PLI	68.00	51.88	13.75	53.63	52.62
		PLI+WPLI	76.00	67.08	10.03	68.43	66.74
		WPLI	58.00	42.40	14.64	51.43	46.05
	Tg-vs-Sc	PLI	56.00	44.28	13.75	42.70	47.79
		PLI+WPLI	59.00	50.26	10.41	47.89	53.67
		WPLI	50.00	39.36	13.67	40.69	40.67
1–30 Hz	Ug-vs-Tg	PLI	60.00	49.16	14.43	50.36	51.17
		PLI+WPLI	87.00	77.18	9.93	79.00	76.38
		WPLI	54.00	40.68	14.40	44.08	39.11
	Ug-vs-Sc	PLI	70.00	58.44	14.42	60.33	59.51
		PLI+WPLI	82.00	75.28	8.73	75.06	76.14
		WPLI	66.00	45.56	13.76	45.39	48.09
	Tg-vs-Sc	PLI	62.00	46.44	15.38	47.11	47.99
		PLI+WPLI	84.00	72.80	9.35	74.86	72.30
		WPLI	54.00	39.52	13.04	34.90	46.93
5 bands fusion	Ug-vs-Tg	PLI	50.00	38.12	14.88	41.93	38.28
		PLI+WPLI	80.00	70.72	10.36	72.35	70.34
		WPLI	40.00	26.88	12.00	31.36	24.78
	Ug-vs-Sc	PLI	64.00	46.56	14.52	49.02	49.07
		PLI+WPLI	75.00	68.04	10.11	68.84	68.23
		WPLI	50.00	38.12	14.58	41.62	38.57
	Tg-vs-Sc	PLI	62.00	45.00	14.89	40.66	52.07
		PLI+WPLI	78.00	69.12	10.21	68.21	71.14
		WPLI	42.00	31.96	12.86	30.30	37.15

To estimate our classification model, we also calculated two important estimation measures in machine learning, sensitivity and specificity, for each classification that was computed for the fusion time windows. As shown in Table 1, the two estimation metrics are also satisfactory, which is consistent with the results of the classification accuracies.

### Brain Network Analysis

To more effectively decode the brain signals related to action intention understanding, we also implemented experiments on brain network analysis with our novel method. In this study, we mainly carried out the experiments from two perspectives: analyzing the difference in the whole brain network between two kinds of action intentions by the rank-sum test and finding the connectivity edges that are obviously uncommon. It is important to note that these two perspectives are based on the dynamic time windows and the two specific frequency bands, alpha and beta.

**Figure 9:**
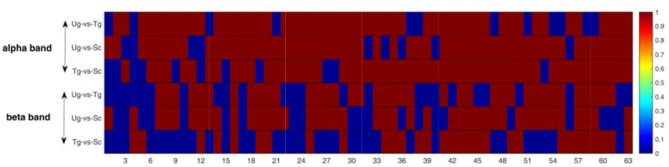
Rank-sum test for the whole brain network. The red domains denote significant differences (*p* < 0.01), and the blue domains represent no significant differences. Each row contains 63 micro-time windows.

Figure 9 shows the results of the pairwise statistical test for the whole brain. We can see that there are many time windows that are significantly different for both the alpha and beta frequency bands (red domains). In general, the alpha band outperforms the beta band in this regard. We can also see that the time windows for the specific ERP components exhibit significant differences (see both Figures 3, 9), e.g., the 19th time window. We first performed the rank-sum test at a significance level of 0.01, and then carried out strict FDR correction at the same significance level.

**Figure 10:**
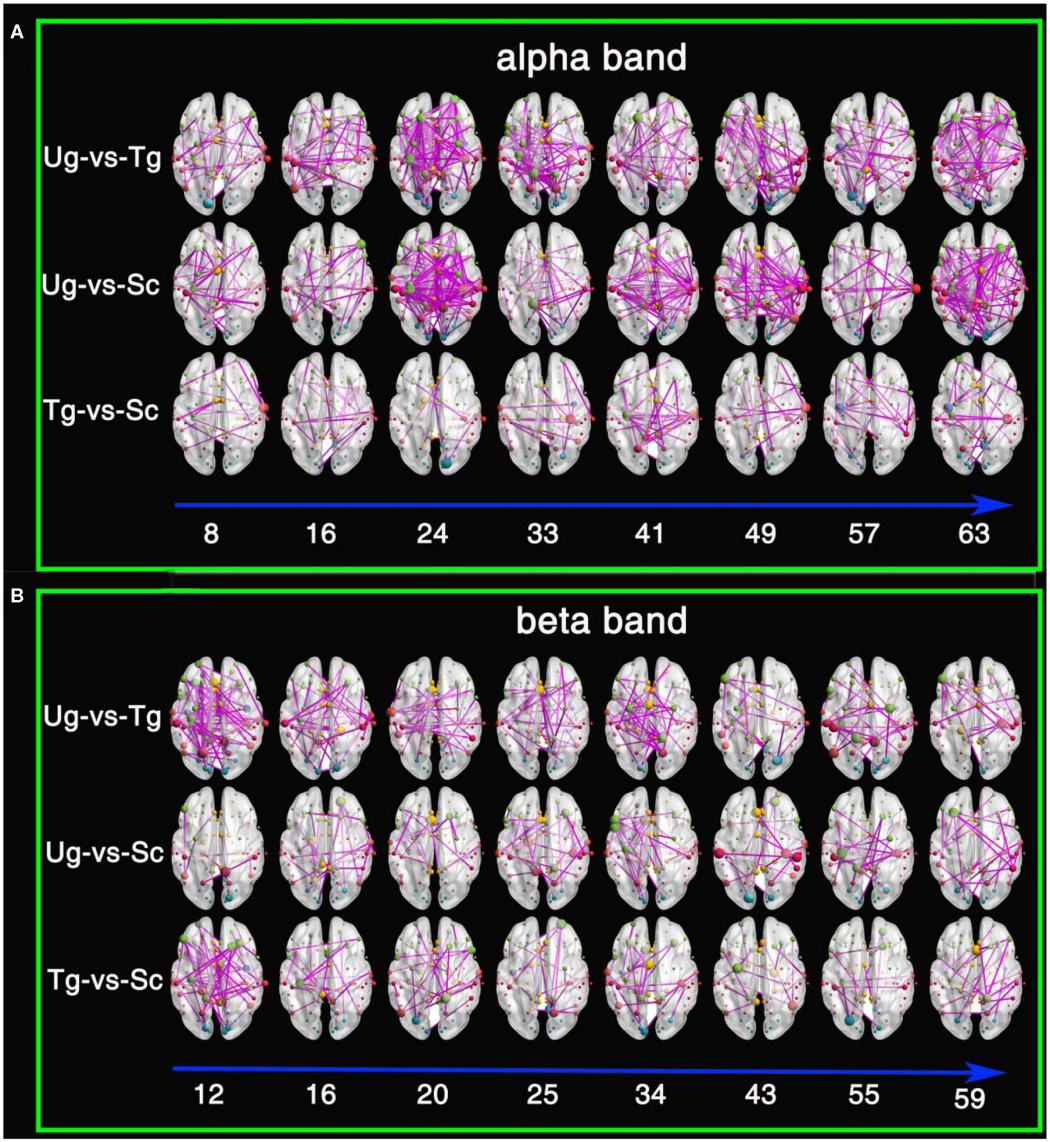
Results of *t*-tests for connectivity edges. **(A)** Comparisons within the alpha frequency band. **(B)** Comparisons within the beta frequency band. The red, yellow, green, cyan, blue, and purple-red nodes are from the temporal lobe, the limbic lobe, the frontal lobe, the occipital lobe, the sub-lobar region, and the parietal lobe, respectively. The size of the node denotes its degree; the larger the size is, the greater the degree. The digits under the blue arrow represent the time window numbers. All connectivity edges were obtained by *t*-test after FDR correction (*p* < 0.01).

Figure 10 shows the results of the *t*-tests for the connectivity edges in multiple time windows. Because we have 63 time windows in total, it is difficult to display all the brain graphs. Hence, for both the alpha and beta bands, we chose 8 time windows that all showed significant differences for all three pairwise intentions from the whole brain network rank-sum test (see Figure 9), for example, the 49th time window in the alpha band and the 55th time window in the beta band. These 8 time windows contain the signals obtained before and after presentation of the formal stimuli (see Figures 1, 3, 5, 10), which can sufficiently satisfy our study task.

From both Figures 10A,B, we can see that there are a greater number of significant connectivity edges in the alpha band, while the beta band is sparser. Additionally, for the alpha band, there are more connectivity edges in the 24th, 41st, 49th, and 63rd time windows than in the others shown. Ug-vs-Tg and Ug-vs-Sc have more connectivity edges than Tg-vs-Sc. In both the alpha and beta frequency bands, many of the larger nodes are found in the frontal, parietal, and occipital lobes. A few larger nodes are found in the limbic lobe and sub-lobar regions.

## Discussion

The main aim of this study was to estimate the performance of the novel method, which uses weighted brain network metrics obtained from both the PLI and WPLI to classify different action intention understanding signals and explore neuronal correlation mechanisms. Some important findings are obtained by the abundance of experimental results. The details of these findings are discussed in detail in the following subsections.

### Analyses of Time Series and Feature Extraction

There are several specific ERP components (e.g., C100, N170) in EEG time series, especially P300 (see Figure 5). This suggests that cognition of action intention understanding is closely correlated with these specific components, which is consistent with other authors' studies (Dong et al., 2010; Ortigue et al., 2010; Deschrijver et al., 2017; Zhang et al., 2017). For the five frequency bands, many significant differences (*p* < 0.05) occur at the time points of these components, which indicates that different intention understandings cause different degrees of brain activity in the same band (Zhang et al., 2017). Aside from these specific components, some significant differences appear at the other time points, e.g., from 400 to 2,000 ms. Beudt and Jacobsen (2015) find that the mentalizing system is activated later than the mirror neuron system, which often responds to early ERP components (e.g., C100 and N170). Ge et al. (2017) and Zhang et al. (2017) also found that the mentalizing system deeply processes the stimulus following the reaction of the mirror neuron system. Thus, there are many significant differences after 400 ms. Overall, Figure 5 proves that our data are clean and reliable and can be used to implement the other experimental tasks.

Our study is mainly based on micro-time windows. Each window was set with a width of 50 ms, and a total of 63 time windows were constructed. In general, the greater the difference between features, the more useful they are (Rodríguez-Bermúdez et al., 2013; Zhang et al., 2015; Miao and Niu, 2016; Ahmadlou and Adeli, 2017; Urbanowicz et al., 2018). From the number of significant “^*^” symbols (*p* < 0.05) shown in Figure 6, we can see that we successfully extracted features that are uncommon between pairs of action intention understanding brain signals. Many significant differences were found between the corresponding time windows for these pairs for the nine graph measures, and each measure has a different efficiency (Kim and Wilhelm, 2008). Both the alpha and beta bands showed satisfactory statistical results, which suggests that action intention understanding is closely correlated with these two bands (Hari, 2006; Ortigue et al., 2010; Avanzini et al., 2012).

### Analyses of Classification Results

Some previous studies on action intention understanding indicate that some differences in the signals occur over time (Ge et al., 2017; Zhang et al., 2017), especially for the special ERP components. Although the classification accuracies in Figure 7 are not very high, the information concerning the change in accuracy is still consistent with previous studies.

The results in Figure 8 and Table 1 suggest that combining PLI with WPLI to produce fusion time windows is a successful method for classifying brain signals. A feasible explanation is that the new approach effectively increases the number of training samples and features, which is extremely important for machine learning (Zhang et al., 2015; Kumar et al., 2016; Miao and Niu, 2016; Pippa et al., 2017; Kang et al., 2018; Urbanowicz et al., 2018). Obviously, compared with PLI or WPLI alone, the combination method has more samples; compared with single time windows, the combination method has more features. Therefore, the new method is more suitable for classification.

Recently, Ahmadlou and Adeli (2017) pointed out that the weighted brain network can retain useful information better than the binary network in neuroscience research. The main reason is that the binary network is sensitive to the threshold (Phillips et al., 2015). Using similar EEG data, other authors' approaches in classifying action intention understanding signals with binary networks (Zhang et al., 2015, 2017) perform worse than our new method, especially for classifying extremely similar stimuli. Notably, these comparisons are indirect. In a word, adopting weighted brain network metrics as the classification features is another good method for improving classification accuracy.

Regarding the accuracies for different frequency bands, we know that the alpha band and whole frequency band (1–30 Hz) yield the best results (see Figure 8). Some previous studies have indicated that major reactions during action observation depend on the alpha and beta bands over the motor areas, especially in the alpha band over the occipito-parietal areas (Hari, 2006; Ortigue et al., 2010; Avanzini et al., 2012). The strong reaction in the alpha band caused a significant difference between the responses to the pairwise stimuli. The *t*-test results in Figures 5, 6 prove this point. Hence, satisfactory accuracies can be obtained, as shown in Figure 8. Why does the whole frequency band also achieve satisfactory result similar to those of the alpha band? We think that this is due to the whole frequency band capturing both alpha and beta band information.

### Analyses of the Dynamic Brain Network

This study is mainly based on multiple micro-time windows, a total of 63 windows from 650 ms before formal stimulation to 2,500 ms after formal stimulation, which can make full use of the signal differences in every time period. Figures 5, 6 present some differences that vary with time, and the results of statistical tests illustrated in both Figures 9, 10 also tell us that action intention understanding is closely correlated with some specific time periods (Ortigue et al., 2010; Ge et al., 2017; Zhang et al., 2017). In Figures 9, 10, compared with the beta band, a greater number of statistically significant *p*-values are illustrated for the alpha band (*p* < 0.01). According to previous studies (Hari, 2006; Ortigue et al., 2010; Avanzini et al., 2012), reactions in the alpha band are more easily induced in response to observing others' actions. Hence, these actions result in the greater number of differences in this band.

Theories concerning the mirror neuron and mental systems indicate that the purposes of others' actions are possibly discriminated by the observers' natural perceptions or indirect inferences (Gallese and Goldman, 1998; Rizzolatti et al., 2001; Rizzolatti and Craighero, 2004; Fogassi et al., 2005; Brass et al., 2007; James, 2008; Liew et al., 2011). Other studies on neuroimaging note that not only mirror neurons but also mental areas take part in action intention understanding (Blakemore and Decety, 2001; De Lang et al., 2008; Van Overwalle and Baetens, 2009; Becchio et al., 2012; Oztop et al., 2013; Catmur, 2015; Tidoni and Candidi, 2016; Ge et al., 2017; Zhang et al., 2017). In the last figure, many large nodes appear in the frontal, occipital, parietal, and temporal cortexes, which suggests that action intention understanding is correlated with these domains. Rizzolatti and Craighero (2004) found that some regions respond when humans observe each other's action behaviors. These regions mainly consist of the inferior parietal lobule, the ventral premotor cortex, the inferior frontal gyrus and the Broca area of the left frontal lobe. Fogassi et al. (2005) find that there is a significant difference in the response in the inferior parietal lobule when monkeys observe actions that look the same but actually denote different intentions. Interestingly, the results in Figure 10 are consistent with this study (Rizzolatti et al., 2001; Rizzolatti and Craighero, 2004; Fogassi et al., 2005), which reconfirms that the mirror neuron system takes part in action intention understanding. Both Figures 10A,B show that the temporal domain has some large nodes under the Ug-vs-Sc and Ug-vs-Sc conditions. Amodio and Frith (2006) and Saxe (2006) note that the mentalizing brain networks mainly consist of the medial prefrontal and temporoparietal junctions and the superior temporal cortexes. Hence, it can be inferred that the action intention understanding in our experiment also involves the mentalizing brain network. Compared with the Ug and Tg stimuli, the action behavior Sc is more abnormal. Therefore, this stimulus can easily cause the significant differences observed in the pairwise Ug-vs-Sc and Tg-vs-Sc comparisons. The essential reason for this is that the mentalizing areas play an important role in responding to abnormal action behaviors (Blakemore and Decety, 2001; Liew et al., 2011; Becchio et al., 2012; Catmur, 2015).

Overall, from the experimental results in Figures 5, 10, we can conclude that both the mirror neuron and mentalizing systems participate in the process of action intention understanding, which is consistent with the results of previous studies (De Lang et al., 2008; Becchio et al., 2012; Catmur, 2015; Ge et al., 2017; Zhang et al., 2017). Whether the relationship between the mirror neuron and mentalizing systems is independent or cooperative in the process of brain activity is debated by many researchers (Van Overwalle and Baetens, 2009; Virji-Babul et al., 2010; Libero et al., 2014; Catmur, 2015; Tidoni and Candidi, 2016; Zhang et al., 2017). Actually, from the experimental results obtained here, we can conclude that the relationship between the mirror neuron and mentalizing systems is cooperative and is capable of encoding the complex dynamical brain signals related to action intention understanding.

### Limitations

Obviously, the new method has many merits in decoding action intention understanding. However, it still needs to be improved. First, an abundance of graph metrics have been proposed for studying network properties in recent years (Kim and Wilhelm, 2008; Rubinov and Sporns, 2010; Gomezpilar et al., 2017). We adopted nine graph measures in total, but other measures (Newman, 2004; Claussen, 2007; Rubinov and Sporns, 2010) might be more useful for exploring action intention understanding. In follow-up research, we will aim to apply new graph measures as classification features. Additionally, we adopted one of the most popular classifiers, SVM, to carry out binary classification. Previous machine learning experience has shown that many classifiers typically obtain different results with the same dataset (Rodríguez-Bermúdez et al., 2013; Miao and Niu, 2016; Urbanowicz et al., 2018). Whether there exist some other classifiers that perform better than the SVM for action intention understanding classification needs to be determined with experimental data. Therefore, this is another future research goal. Finally, which system is the most important in the process of an agent observing another's actions, i.e., whether the mirror neuron or the mentalizing system dominates the action intention understanding, has not been thoroughly decoded (Becchio et al., 2012; Marsh et al., 2014; Catmur, 2015; Tidoni and Candidi, 2016; Ge et al., 2017; Pomiechowska and Csibra, 2017). Therefore, a more comprehensive study on the neuronal mechanism underlying action intention understanding needs to be implemented in the future.

## Conclusion

In summary, this study highlights a combination method that decodes the brain signals related to action intention understanding by combining the weighted brain network metrics of both the PLI and WPLI. Sample and feature fusion efficiently improve the classification accuracy, especially for similar action intention stimuli. Compared with the low frequency and beta bands, the differences in the action intention understanding brain signals are more obvious in the alpha band. The new approach can be universally applied for many studies. Brain activity signals collected by MRI, fMRI, MEG, and NIRS can be analyzed with our novel method. Other psychological and cognitive behavior data (e.g., mathematical deduction and emotion recognition) analyses can also use the new method. Overall, it has the advantage of generality.

## Data Availability Statement

The raw data supporting the conclusions of this article will be made available by the authors, without undue reservation, to any qualified researcher.

## Ethics Statement

The studies involving human participants were reviewed and approved by the Academic Committee of the School of Biological Sciences and Medical Engineering, Southeast University, China. The patients/participants provided their written informed consent to participate in this study.

## Author Contributions

XX conceived and designed the study and wrote the manuscript. ZY, TM, NL, and HF collected and analyzed the data. HW and XL reviewed and edited the manuscript. All authors read and approved the manuscript.

## Conflict of Interest

The authors declare that the research was conducted in the absence of any commercial or financial relationships that could be construed as a potential conflict of interest.

## References

[B1] Ahmadlou M. Adeli H. (2017). Complexity of weighted graph: a new technique to investigate structural complexity of brain activities with applications to aging and autism. Neurosci. Lett. 650, 103–108. 10.1016/j.neulet.2017.04.009 28414133

[B2] Amodio D. M. Frith C. D. (2006). Meeting of minds: the medial frontal cortex and social cognition. Nat. Rev. Neurosci. 7, 268–277. 10.1038/nrn1884 16552413

[B3] Arnaud D. Scott M. (2004). EEGLAB: an open source toolbox for analysis of single-trial EEG dynamics including independent component analysis. J. Neurosci. Methods 134, 9–21. 10.1016/j.jneumeth.2003.10.009 15102499

[B4] Avanzini P. Fabbridestro M. Volta R. D. Daprati E. Rizzolatti G. Cantalupo G. (2012). The dynamics of sensorimotor cortical oscillations during the observation of hand movements: an EEG study. PloS ONE 7:e37534. 10.1371/journal.pone.0037534 22624046 PMC3356327

[B5] Baccalá L. A. Sameshima K. (2001). Partial directed coherence: a new concept in neural structure determination. Biol. Cybern. 84, 463–474. 10.1007/PL00007990 11417058

[B6] Barrat A. Barthelemy M. Pastor-Satorras R. Vespignani A. (2004). The architecture of complex weighted networks. Proc. Natl. Acad. Sci. U.S.A. 101, 3747–3752. 10.1073/pnas.0400087101 15007165 PMC374315

[B7] Becchio C. Cavallo A. Begliomini C. Sartori L. Feltrin G. Castiello U. (2012). Social grasping: from mirroring to mentalizing. Neuroimage 61, 240–248. 10.1016/j.neuroimage.2012.03.013 22440652

[B8] Beudt S. Jacobsen T. (2015). On the role of mentalizing processes in aesthetic appreciation: an ERP study. Front. Hum. Neurosci. 9:600. 10.3389/fnhum.2015.00600 26617506 PMC4643139

[B9] Blakemore S. J. Decety J. (2001). From the perception of action to the understanding of intention. Nat. Rev. Neurosci. 2, 561–567. 10.1038/35086023 11483999

[B10] Bockbrader M. A. Francisco G. Lee R. Olson J. Solinsky R. Boninger M. L. (2018). Brain computer interfaces in rehabilitation medicine. PMR 10, 233–243. 10.1016/j.pmrj.2018.05.028 30269808

[B11] Bonini L. Ferrari P. F. Fogassi L. (2013). Neurophysiological bases underlying the organization of intentional actions and the understanding of others' intention. Conscious. Cogn. 22, 1095–1104. 10.1016/j.concog.2013.03.001 23545395

[B12] Brass M. Schmitt R. M. Spengler S. Gergely G. (2007). Investigating action understanding: inferential processes versus action simulation. Curr. Biol. 17, 2117–2121. 10.1016/j.cub.2007.11.057 18083518

[B13] Brune C. Woodward A. L. (2007). Social cognition and social responsiveness in 10-month-old infants. J. Cogn. Dev. 8, 133–158. 10.1080/15248370701202331

[B14] Cacioppo S. Juan E. Monteleone G. (2017). Predicting intentions of a familiar significant other beyond the mirror neuron system. Front. Behav. Neurosci. 11:155. 10.3389/fnbeh.2017.00155 28890691 PMC5574908

[B15] Cacippo J. T. Berntson G. G. Decety J. (2010). Social neuroscience and its relationship to social psychology. Soc. Cogn. 28, 675–685. 10.1521/soco.2010.28.6.675 24409007 PMC3883133

[B16] Carter E. J. Hodgins J. K. Rakison D. H. (2011). Exploring the neural correlates of goal-directed action and intention understanding. Neuroimage 54, 1634–1642. 10.1016/j.neuroimage.2010.08.077 20832476

[B17] Casteel M. A. (2011). The influence of motor simulations on language comprehension. Acta Psychol. 138, 211–218. 10.1016/j.actpsy.2011.06.006 21763635

[B18] Catmur C. (2015). Understanding intentions from actions: direct perception, inference, and the roles of mirror and mentalizing systems. Conscious. Cogn. 36, 426–433. 10.1016/j.concog.2015.03.012 25864592

[B19] Cignetti F. Vaugoyeau M. Decker L. M. Grosbras M. H. Girard N. Chaix Y.. (2018). Brain network connectivity associated with anticipatory postural control in children and adults. Cortex 108, 210–221. 10.1016/j.cortex.2018.08.013 30248609

[B20] Claussen J. C. (2007). Offdiagonal complexity: a computationally quick complexity measure for graphs and networks. Phys. A 375, 365–373. 10.1016/j.physa.2006.08.067

[B21] Cole E. J. Barraclough N. E. (2018). Timing of mirror system activation when inferring the intentions of others. Brain Res. 1700, 109–117. 10.1016/j.brainres.2018.07.015 30016631

[B22] Cole E. J. Barraclough N. E. Enticott P. G. (2018). Investigating mirror system (MS) activity in adults with ASD when inferring others' intentions using both TMS and EEG. J. Autism Dev. Disord. 48, 2350–2367. 10.1007/s10803-018-3492-2 29453710 PMC5996018

[B23] De Lang F. P. Spronk M. Willems R. M. Toni I. Bekkering H. (2008). Complementary systems for understanding action intentions. Curr. Biol. 18, 454–457. 10.1016/j.cub.2008.02.057 18356050

[B24] Deschrijver E. Wiersema J. R. Brass M. (2017). The influence of action observation on action execution: Dissociating the contribution of action on perception, perception on action, and resolving conflict. Cogn. Affect. Behav. Neurosci. 17, 381–393. 10.3758/s13415-016-0485-5 28000082

[B25] Dindo H. Lopresti L. Lacascia M. Chella A. Dedić R. (2017). Hankelet-based action classification for motor intention recognition. Robot. Auton. Syst. 94, 120–133. 10.1016/j.robot.2017.04.003

[B26] Dong G. Hu Y. Zhou H. (2010). Event-related potential measures of the intending process: Time course and related ERP components. Behav. Brain Funct. 6, 15–15. 10.1186/1744-9081-6-15 20178644 PMC2848189

[B27] Fogassi L. Ferrari P. F. Gesierich B. Rozzi S. Chersi F. Rizzolatti G. (2005). Parietal lobe: from action organization to intention understanding. Science 308, 662–667. 10.1126/science.1106138 15860620

[B28] Gallese V. Goldman A. (1998). Mirror neurons and the simulation theory of mind of mind-reading. Trends Cogn. Sci. 2, 493–501. 10.1016/S1364-6613(98)01262-5 21227300

[B29] Ge S. Ding M. Y. Zhang Z. Lin P. Gao J. F. Wang R. M. (2017). Temporal-spatial features of intention understanding based on EEG-fNIRS bimodal measurement. IEEE Access 5, 14245–14258. 10.1109/ACCESS.2017.2723428

[B30] Gomezpilar J. Poza J. Bachiller A. Gómez C. Núñez P. Lubeiro A.. (2017). Quantification of graph complexity based on the edge weight distribution balance: application to brain networks. Int. J. Neural Syst. 28:1750032. 10.1142/S0129065717500320 28691561

[B31] Hari R. (2006). Action-perception connection and the cortical mu rhythm. Prog. Brain Res. 159, 253–260. 10.1016/S0079-6123(06)59017-X 17071236

[B32] Humphries M. D. Gurney K. (2008). Network ‘small-world-ness’: a quantitative method for determining canonical network equivalence. PLoS ONE 3:e0002051. 10.1371/journal.pone.0002051 18446219 PMC2323569

[B33] Isoda M. (2016). Understanding intentional actions from observers' viewpoints: a social neuroscience perspective. Neurosci. Res. 112, 1–9. 10.1016/j.neures.2016.06.008 27393254

[B34] James M. K. (2008). Action observation: inferring intentions without mirror neurons. Curr. Biol. 18, 32–33. 10.1016/j.cub.2007.11.008 18177711

[B35] Kang J. Zhou T. Han J. Li X. (2018). EEG-based multi-feature fusion assessment for autism. J. Clin. Neurosci. 56, 101–107. 10.1016/j.jocn.2018.06.049 30318070

[B36] Kaschak M. P. Madden C. J. Therriault D. J. Yaxley R. H. Aveyard M. Blanchard A. A.. (2010). Perception of motion affects language processing. Cognition 30, 733–744. 10.1207/s15516709cog0000_54 15617669

[B37] Kim J. Wilhelm T. (2008). What is a complex graph? Phys. A 387, 2637–2652. 10.1016/j.physa.2008.01.015

[B38] Kumar N. V. Pratheek K. V. V. Govindaraju K. N. Guru D. S. (2016). Features fusion for classification of logos. Proc. Comput. Sci. 85, 370–379. 10.1016/j.procs.2016.05.245

[B39] Lachaux J. P. Rodriguez E. Martinerie J. Varela F. J. (1999). Measuring phase synchrony in brain signals. Hum. Brain Mapp. 8, 194–208. 10619414 10.1002/(SICI)1097-0193(1999)8:4<194::AID-HBM4>3.0.CO;2-C PMC6873296

[B40] Latora V. Marchiori M. (2001). Efficient behavior of small-world networks. Phys. Rev. Lett. 87:198701 10.1103/PhysRevLett.87.198701 11690461

[B41] Libero L. E. Maximo J. O. Deshpande H. D. Klinger L. G. Klinger M. R. Kana R. K. (2014). The role of mirroring and mentalizing networks in mediating action intentions in autism. Mol. Autism 5:50. 10.1186/2040-2392-5-50 25352976 PMC4210608

[B42] Liew S. L. Han S. Aziz-Zadeh L. (2011). Familiarity modulates mirror neuron and mentalizing regions during intention understanding. Hum. Brain Mapp. 32, 1986–1997. 10.1002/hbm.21164 20882581 PMC6870503

[B43] Liu H. Zheng W. M. Sun G. Shi Y. Leng Y. Lin P. (2017). Action understanding based on a combination of one-versus-rest and one-versus-one multi-classification methods. Int. Congress Image Signal Process Biomed. Eng. Inform. 10, 1–5. 10.1109/CISP-BMEI.2017.8302159

[B44] Marsh L. E. Mullett T. L. Ropar D. Hamilton A. F. (2014). Responses to irrational actions in action observation and mentalising networks of the human brain. Neuroimage 103, 81–90. 10.1016/j.neuroimage.2014.09.020 25241085

[B45] Mcfarland D. J. Wolpaw J. R. (2017). EEG-based brain-computer interfaces. Curr. Opin. Biomed. Eng. 4:194. 10.1016/j.cobme.2017.11.004 29527584 PMC5839510

[B46] Miao J. Niu L. (2016). A survey on feature selection. Procedia Comput. Sci. 91, 919–926. 10.1016/j.procs.2016.07.111

[B47] Newman M. E. (2004). Fast algorithm for detecting community structure in networks. Phys. Rev. E Stat. Nonlin. Soft Matter Phys. 69:066133. 10.1103/PhysRevE.69.066133 15244693

[B48] Niso G. Bruña R. Pereda E. Gutiérrez R. Bajo R. Maestú F.. (2013). HERMES: towards an integrated toolbox to characterize functional and effective brain connectivity. Neuroinformatics 11, 405–434. 10.1007/s12021-013-9186-1 23812847

[B49] Nolte G. Bai O. Wheaton L. Mari Z. Vorbach S. Hallett M. (2004). Identifying true brain interaction from EEG data using the imaginary part of coherency. Clin. Neurophysiol. 115, 2292–2307. 10.1016/j.clinph.2004.04.029 15351371

[B50] Ofner P. Schwarz A. Pereira J. Müller-Putz G. R. (2017). Upper limb movements can be decoded from the time-domain of low-frequency EEG. PloS ONE 12:e0182578. 10.1371/journal.pone.0182578 28797109 PMC5552335

[B51] Onnela J. P. Jari S. K. János K. Kimmo K. (2005). Intensity and coherence of motifs in weighted complex networks. Phys. Rev. E: Stat. Nonlin. Soft Matter Phys. 71:065103. 10.1103/PhysRevE.71.065103 16089800

[B52] Ortigue S. Sinigaglia C. Rizzolatti G. Grafton S. T. (2010). Understanding actions of others: the electrodynamics of the left and right hemispheres. A high-density EEG neuroimaging study. PLoS ONE 5:e12160. 10.1371/journal.pone.0012160 20730095 PMC2921336

[B53] Ouden-Den H. E. Frith U. Frith C. Blakemore S. J. (2005). Thinking about intentions. Neuroimage 28, 787–796. 10.1016/j.neuroimage.2005.05.001 15964210

[B54] Oztop E. Kawato M. Arbib M. A. (2013). Mirror neurons: functions, mechanisms and models. Neurosci. Lett. 540, 43–55. 10.1016/j.neulet.2012.10.005 23063951

[B55] Pereira J. Ofner P. Schwarz A. Sburlea A. L. Mullerputz G. R. (2017). EEG neural correlates of goal-directed movement intention. Neuroimage 149, 129–140. 10.1016/j.neuroimage.2017.01.030 28131888 PMC5387183

[B56] Phillips D. J. Mcglaughlin A. Ruth D. Jager L. R. Soldan A. (2015). Graph theoretic analysis of structural connectivity across the spectrum of alzheimer's disease: the importance of graph creation methods. Neuroimage Clin. 7, 377–390. 10.1016/j.nicl.2015.01.007 25984446 PMC4429220

[B57] Pippa E. Zacharaki E. I. Koutroumanidis M. Megalooikonomou V. (2017). Data fusion for paroxysmal events' classification from EEG. J. Neurosci. Methods 275, 55–65. 10.1016/j.jneumeth.2016.10.004 27845151

[B58] Pomiechowska B. Csibra G. (2017). Motor activation during action perception depends on action interpretation. Neuropsychologia 105:84. 10.1016/j.neuropsychologia.2017.01.032 28189494 PMC5447367

[B59] Rizzolatti G. Craighero L. (2004). The mirro-neuron system. Annu. Rev. Neurosci. 27, 169–192. 10.1146/annurev.neuro.27.070203.144230 15217330

[B60] Rizzolatti G. Fogassi L. Gallese V. (2001). Neurophysiological mechanisms underlying the understanding and imitation of action. Annu. Rev. Neurosci. 2, 661–670. 10.1038/35090060 11533734

[B61] Rodríguez-Bermúdez G. García-Laencina P. J. Roca-González J. Roca-Dorda J. (2013). Efficient feature selection and linear discrimination of EEG signals. Neurocomputing 115, 161–165. 10.1016/j.neucom.2013.01.001

[B62] Rubinov M. Sporns O. (2010). Complex network measures of brain connectivity: uses and interpretations. Neuroimage 52, 1059–1069. 10.1016/j.neuroimage.2009.10.003 19819337

[B63] Satpute A. B. Fenker D. B. Waldmann M. R. Tabibnia G. Holyoak K. J. Lieberman M. D. (2005). An f-MRI study of causal judgements. Eur. J. Neurosci. 22, 1233–1238. 10.1111/j.1460-9568.2005.04292.x 16176366

[B64] Saxe R. (2006). Uniquely human social cognition. Curr. Opin. Neurobiol. 16, 235–239. 10.1016/j.conb.2006.03.001 16546372

[B65] Schreiber T. (2000). Measuring information transfer. Phys. Rev. Lett. 85, 461–464. 10.1103/PhysRevLett.85.461 10991308

[B66] Stam C. J. Dijk B. W. (2002). Synchronization likelihood: an unbiased measure of generalized synchronization in multivariate data sets. Phys. D 163, 236–251. 10.1016/S0167-2789(01)00386-4

[B67] Stam C. J. Nolte G. Daffertshofer A. (2007). Phase lag index: assessment of functional connectivity from multi channel EEG and MEG with diminished bias from common sources. Hum. Brain Mapp. 28, 1178–1193. 10.1002/hbm.20346 17266107 PMC6871367

[B68] Tidoni E. Candidi M. (2016). Commentary: understanding intentions from actions: direct perception, inference, and the roles of mirror and mentalizing systems. Front. Behav. Neurosci. 10:13. 10.3389/fnbeh.2016.00013 26903829 PMC4749676

[B69] Urbanowicz R. J. Meeker M. Cava W. Olson R. S. Moore J. H. (2018). Relief-based feature selection: introduction and review. J. Biomed. Inform. 85, 189–203. 10.1016/j.jbi.2018.07.014 30031057 PMC6299836

[B70] Van Overwalle F. V. Baetens K. (2009). Understanding others' actions and goals by mirror and mentalizing systems: a meta-analysis. Neuroimage 48, 564–584. 10.1016/j.neuroimage.2009.06.009 19524046

[B71] Vecchio F. Miraglia F. Maria-Rossini P. (2017). Connectome: graph theory application in functional brain network architecture. Clin. Neurophysiol. Pract. 2, 206–213. 10.1016/j.cnp.2017.09.003 30214997 PMC6123924

[B72] Vinck M. Oostenveld R. van Wingerden M. Battaglia F. Pennartz C. M. A. (2011). An improved index of phase-synchronization for electrophysiological data in the presence of volume-conduction, noise and sample-size bias, Neuroimage 55, 1548–1565. 10.1016/j.neuroimage.2011.01.055 21276857

[B73] Virji-Babul N. Moiseev A. Cheung T. Weeks D. Cheyne D. Ribary U. (2010). Spatial-temporal dynamics of cortical activity underlying reaching and grasping. Hum. Brain Mapp. 31, 160–171. 10.1002/hbm.20853 19593776 PMC6871227

[B74] Watts D. J. Strogatz S. H. (1998). Collective dynamics of ‘small-world’ networks. Nature 393, 440–442. 10.1038/30918 9623998

[B75] Zhang L. Gan J. Q. Zheng W. M. Wang H. X. (2017). Spatiotemporal phase synchronization in adaptive reconfiguration from action observation network to mentalizing network for understanding other's action intention. Brain Topogr. 31, 1–21. 10.1007/s10548-017-0614-7 29264681

[B76] Zhang Z. Yang Q. Leng Y. Yang Y. Ge S. (2015). Classification of intention understanding using EEG-NIRS bimodal system. Int. Comput. Conf. Wavelet Act. Med. Technol. Inform. Proc. 12, 67–70. 10.1109/ICCWAMTIP.2015.7493908

